# GBM-associated mutations and altered protein expression are more common in young patients

**DOI:** 10.18632/oncotarget.11617

**Published:** 2016-08-25

**Authors:** Sherise D. Ferguson, Joanne Xiu, Shiao-Pei Weathers, Shouhao Zhou, Santosh Kesari, Stephanie E. Weiss, Roeland G. Verhaak, Raymond J. Hohl, Geoffrey R. Barger, Sandeep K. Reddy, Amy B. Heimberger

**Affiliations:** ^1^ Department of Neurosurgery, Biostatistics, The University of Texas M.D. Anderson Cancer Center, Houston, TX 77030, USA; ^2^ Department of Neuro-Oncology, The University of Texas M.D. Anderson Cancer Center, Houston, TX 77030, USA; ^3^ Department of Biostatistics, The University of Texas M.D. Anderson Cancer Center, Houston, TX 77030, USA; ^4^ Caris Life Sciences, Phoenix, AZ 85040, USA; ^5^ Department of Translational Neuro-Oncology and Neurotherapeutics, Pacific Neuroscience Institute and John Wayne Cancer Institute at Providence Saint John's Health Center, Santa Monica, CA 90404, USA; ^6^ Fox Chase Cancer Center, Philadelphia, PA 19111, USA; ^7^ Department of Genome Medicine, The University of Texas M.D. Anderson Cancer Center, Houston, TX 77054, USA; ^8^ Penn State Hershey Cancer Institute, Hershey, PA 17033, USA; ^9^ Department of Neurology, Wayne State University, School of Medicine, Karmanos Cancer Center, Detroit, MI 48201, USA

**Keywords:** GBM, mutational analysis, DNA sequencing

## Abstract

**Background:**

Geriatric glioblastoma (GBM) patients have a poorer prognosis than younger patients, but *IDH1/2* mutations (more common in younger patients) confer a favorable prognosis. We compared key GBM molecular alterations between an elderly (age ≥ 70) and younger (18 < = age < = 45) cohort to explore potential therapeutic opportunities.

**Results:**

Alterations more prevalent in the young GBM cohort compared to the older cohort (*P* < 0.05) were: overexpression of ALK, RRM1, TUBB3 and mutation of *ATRX, BRAF, IDH1,* and *TP53*. However, *PTEN* mutation was significantly more frequent in older patients. Among patients with wild-type *IDH1/2* status, TOPO1 expression was higher in younger patients, whereas MGMT methylation was more frequent in older patients. Within the molecularly-defined IDH wild-type GBM cohort, younger patients had significantly more mutations in *PDGFRA, PTPN11, SMARCA4, BRAF* and *TP53*.

**Methods:**

GBMs from 178 elderly patients and 197 young patients were analyzed using DNA sequencing, immunohistochemistry, *in situ* hybridization, and MGMT-methylation assay to ascertain mutational and amplification/expressional status.

**Conclusions:**

Significant molecular differences occurred in GBMs from elderly and young patients. Except for the older cohort's more frequent *PTEN* mutation and MGMT methylation, younger patients had a higher frequency of potential therapeutic targets.

## INTRODUCTION

Aging is considered to confer the greatest risk for the development of a malignancy. Presumably the accumulated lifetime exposure to DNA-damaging agents and age-related impaired DNA repair and replicative capacity would demonstrate a positive correlation of tumor mutation rate and age [1, 2]. Precluding the former theory, even if genomic changes occur during DNA replication by chance and are based on the number of stem cell divisions and not related directly to DNA damage/repair, there would still be an association with age [3]. Overall, studies have revealed that there are approximately 140 genes that can promote tumorigenesis across the spectrum of tumors, with each tumor typically containing two to eight of these driver mutations conferring selective growth advantage [4]. Based on the aforementioned premise, older patients would be predicted to be more likely to have an actionable mutation for targeted therapeutics. Tumor mutations may also correlate with clinical responses to immune therapeutics such as immune checkpoint inhibitors [5, 6] however, this has yet to be validated. Moreover, it is still somewhat controversial as to the role of immune checkpoint blockade in GBM treatment as no clinical studies to date confirm efficacy. GBM (glioblastoma) is the most common primary brain tumor of adulthood, and younger age is a positive prognostic factor [7]. This association has been attributed to a higher pretreatment KPS score, a greater likelihood of aggressive therapeutic interventions, increased eligibility for clinical trials, and a more robust social support system [8]. A positive correlation between mutation frequency and age among GBM patients has recently been demonstrated [9]. Age-related molecular signatures in GBM patients have been previously documented—specifically *IDH1* alterations, which are not only prognostic but which also molecularly define primary versus secondary GBM [10–14]. In this study, we specifically analyzed genes that have been previously shown to play a crucial role within the context of GBM and their association with age. We compared these molecular profiles, obtained in a Clinical Laboratory Improvement Amendments (CLIA)-certified laboratory, of a large cohort of GBM samples obtained from younger (18–45 years) and older patients (> 70 years). Identification of specific molecular alterations may provide an opportunity to identify patients likely to benefit from targeted therapies or define cohorts of patients who may benefit from more comprehensive molecular profiling.

## RESULTS

### Study population

A total of 375 adult GBM samples comprised the data set that was submitted to a commercial company conducting molecular profiling. Specimens were procured at various times during the disease course. The study group was dichotomized into an older cohort of patients aged 70 years or greater (*N* = 178; 47%) and a younger cohort of patients less than 45 years old (*N* = 197; 53%). Patients between the ages of 45 and 70, those with astrocytomas of WHO grade III or below, or with oligodendrogliomas, were excluded. Distribution of the sexes was well-balanced in the dichotomized age groups: 38% (75/197) and 37% (67/178) were female in the younger and older cohorts, respectively. Patient treatment history and response were not part of the data collection because this is a commercial repository.

### Age association of GBM-specific protein expression

An analysis of 18 biomarkers by IHC revealed that regardless of age, TUBB3 was most frequently expressed at 85% of cases, followed by PTEN and EGFR at 78.5% and 77.5%, respectively (Table 1), consistent with prior reports [15]. Age-dependent differential expression of three proteins was found: ALK, RRM1, and TUBB3. Specifically, 29% (7/24) of patients less than 45 years old expressed ALK versus only 4.2% of elderly patients (*P* = 0.0479). RRM1 was expressed in 48% (65/136) of younger patients compared with 31.6% (37/117) of elderly patients (*P* = 0.0103). TUBB3 showed very high expression in the younger group, staining positively in 93% (94/101) of samples relative to 76% (69/90) in the elderly group (*P* = 0.0018). There were no age-dependent differences in the expression of PD-1, PD-L1, MGMT, EGFR, or PTEN.

**Table 1:** Immunohistochemical analysis of all GBMs

	Patient age < 45			Patient age ≥ 70		
Gene	Positive	Total	%	Positive	Total	%
**ALK**	7	24	29.2^*^	1	24	4.2
cMET	6	117	5.1	2	108	1.9
EGFR	51	65	78.5	42	55	76.4
ERCC1	37	98	37.8	32	86	37.2
MGMT	7	81	8.6	3	60	5
PD-1	27	64	42.2	29	54	53.7
PD-L1	13	69	18.8	5	60	8.3
PGP	8	139	5.8	7	131	5.3
PR	11	143	7.7	7	134	5.2
PTEN	142	180	78.9	118	151	78.1
**RRM1**	65	136	47.8^*^	37	117	31.6
SPARCm	21	127	16.5	14	104	13.5
SPARCp	16	136	11.8	6	111	5.4
TLE3	39	108	36.1	36	100	36
TOP2A	90	148	60.8	69	130	53.1
TOPO1	96	167	57.5	73	148	49.3
TS	89	162	54.9	70	140	50
**TUBB3**	94	101	93.1^*^	69	90	76.7

* indicates *P* value < 0.05.

List of abbreviations: ALK: anaplastic lymphoma kinase; cMET: MET or hepatocyte growth factor receptor; EGFR: epidermal growth factor receptor; ERCC1: excision repair cross-complementation group 1; MGMT: O^6^-methylguanine-DNA methyltransferase; PD-1: programmed cell death 1; PD-L1: programmed cell death ligand 1; PGP: P-glycoprotein; PR: progesterone receptor; PTEN: phosphatase and tensin homolog; RRM1: ribonucleotide reductase subunit M1; SPARC: secreted protein acidic and rich in cysteine; TLE3: transducing-like enhancer of split 3; TOP2A: topoisomerase II alpha; TOPO1: topoisomerase I; thymidylate synthase; TUBB3: class III beta-tubulin.

### Mutations segregate by age in GBM patients

Consistent with prior reports, 1p/19q codeletion is a rare event in GBM and was only found in the younger cohort (*P* = 0.0566) [15, 16]. Either CISH or FISH was used to evaluate gene amplification of *cMET*, revealing an amplification rate of less than 2% of GBM cases in both the young and elderly patient groups consistent with previous literature [15]. The large deletion of exons 2–7 on the extracellular domain of *EGFR* (*EGFRvIII*) was tested by fragment analysis, and there was no significant difference in *EGFRvIII* expression between older and younger age groups (14% versus 12%, respectively) in 131 samples.

A panel of well-known, pan-cancer-related genes was constructed from a dataset of 592 total mutations (Table 2). Because this is a commercial repository, physicians’ requests for specific alterations and profiling were limited to subsets of patients. The most frequently mutated gene in the younger subgroup was *ATRX*, in 66.7% (6/9 cases) followed by *TP53*, in 56.6% (56/99) of cases. In the elderly cohort, the most frequently altered genes were *PTEN* and *TP53*, with each occurring in 25.8% of cases (24/93 and 25/97, respectively). Younger patients were more likely than older ones to have a mutation in *TP53* (56.6% versus 25.8%, respectively; *P* < 0.0001) and *BRAF* (9.3% versus 1.7%, respectively; *P* = 0.0117). As expected, younger patients were also more likely than older ones to carry *IDH1* mutations (26% versus 3.1%, respectively; *P* < 0.0001). Interestingly, the only mutation more frequently mutated in elderly patients than younger ones was *PTEN* (26% versus 12.5%, respectively; *P* = 0.0258).

**Table 2:** Mutational analysis (sequencing): All GBMs

	Patient age < 45			Patient age ≥ 70		
Gene	Positive	Total tested	%	Positive	Total tested	%
ABL1	2	98	2	1	93	1.1
AKT1	0	100	0	1	98	1
ALK	0	100	0	0	98	0
Androgen Receptor	0	10	0	0	10	0
APC	4	100	4	7	98	7.1
ARAF	0	10	0	0	10	0
ATM	1	100	1	5	97	5.2
**ATRX**	6	9	66.7^*^	1	9	11.1
BAP1	0	10	0	1	10	10
**BRAF**	12	129	9.3^*^	2	118	1.7
BRCA1	2	59	3.4	2	39	5.1
BRCA2	4	59	6.8	2	38	5.3
CDK4	0	10	0	0	10	0
CDKN2A	1	7	14.3	1	8	12.5
CHEK1	0	10	0	0	10	0
CHEK2	0	10	0	0	10	0
cKIT	5	107	4.7	2	107	1.9
cMET	2	100	2	0	98	0
CSF1R	3	100	3	0	98	0
CTNNB1	1	100	1	0	98	0
DDR2	0	10	0	0	10	0
EGFR	6	101	5.9	7	99	7.1
ERBB2	0	97	0	0	94	0
ERBB3	0	10	0	0	10	0
FGFR1	0	100	0	0	98	0
FGFR2	2	99	2	0	98	0
FGFR3	1	10	10	0	10	0
FLT3	0	99	0	1	98	1
GNA11	2	92	2.2	0	86	0
GNAQ	0	82	0	0	81	0
GNAS	1	100	1	0	98	0
HRAS	0	88	0	0	80	0
**IDH1**	26	100	26^*^	3	98	3.1
IDH2	1	73	1.4	0	61	0
JAK2	0	100	0	1	98	1
KDR	0	100	0	1	98	1
KRAS	4	119	3.4	2	116	1.7
MEK1	0	10	0	0	10	0
MEK2	0	10	0	0	10	0
MLH1	2	99	2	0	98	0
MPL	0	99	0	0	95	0
NF1	4	10	40	2	10	20
NOTCH1	0	97	0	0	98	0
NRAS	0	110	0	2	106	1.9
NTRK1	0	10	0	0	10	0
PDGFRA	5	99	5.1^^^	0	97	0
PDGFRB	0	10	0	0	10	0
PIK3CA	14	120	11.7	6	110	5.5
PTCH1	0	10	0	1	10	10
**PTEN**	12	96	12.5	24	93	25.8^*^
PTPN11	6	99	6.1	1	98	1
RAF1	2	10	20	0	10	0
RET	0	93	0	2	91	2.2
ROS1	0	10	0	1	10	10
SMARCA4	3	10	30	0	10	0
SMO	1	87	1.1	1	83	1.2
**TP53**	56	99	56.6^*^	25	97	25.8
VHL	0	91	0	0	89	0
WTI	0	9	0	1	10	10

* indicates *P* value < 0.05;

^ indicates *P* value > 0.05 but < 0.10.

### Mutation and age associations within molecularly-defined GBM

Because the field is evolving from a definition of GBM based on histological characteristics to one based on molecular signatures, we performed a secondary analysis in GBM patients who had wild-type *IDH1* status, in order to assess for age variation in biomarker expression (Table 3). Among the IHC markers, the association of TUBB3 with younger age was confirmed in the *IDH1* wild-type GBM population, but this association no longer held for RRM1 and ALK (Table 3). Specifically, TUBB3 was highly expressed in younger patients relative to older ones (91% [61/67] versus 77% [64/83], respectively; *P* = 0.0268). Similarly, there was an increased incidence of expression of TOPO1 in younger patients relative to older ones (63.4% [45/71] versus 46.6% [41/88], respectively; *P* = 0.0406) that was a noted trend upon analysis of the histologically-defined GBM cases (Table 1). There was no age-dependent difference in expression in PD-1, PD-L1 or PTEN. Overall 1p19q codeletion occurred in less than 10% of the *IDH* wild-type GBM population, and again, only in the younger population. The enrichment of *BRAF*, *PDGFRA*, and *TP53* mutations in the younger patient cohort was also statistically different in the molecularly-defined subset GBM patients (Table 4). Mutations in several other genes such as *PTPN11* and *SMARCA4* also showed consistent trends of enrichment in the younger cohort between these two subsets. (Table 1 and Table 4). There was a higher frequency of *BRAF* mutations in the younger *IDH* wild-type patients compared with older patients (12.5% [9/72] versus 1.1% [1/95], respectively; *P* = 0.0053). No patients in the elderly cohort had mutations in *SMARCA4* or *PDGFRA*; however, 50% (3/6) of younger patients showed a *SMARCA4* mutation (*P* = 0.0357) and 4.2% (3/71) carried a *PDGFRA* mutation (*P* = 0.0348). Younger patients had more frequent mutations in *PTPN11* (7% [5/71] versus 1.1% [1/95], respectively; *P* = 0.0437). Lastly, a higher frequency of *PTEN* mutations was again observed in geriatric patients with molecularly defined GBM, but this did not reach statistical significance.

**Table 3:** Immunohistochemical analysis: IDH1/2 wild type GBMs

	Age < 45			Age ≥ 70		
Gene	Positive	Total	%	Positive	Total	%
ALK	6	22	27.3^^^	1	22	4.5
cMET	2	64	3.1	1	80	1.3
EGFR	32	38	84.2	36	47	76.6
ERCC1	7	31	22.6	15	36	41.7
MGMT	1	9	11.1	1	5	20
PD-1	22	47	46.8	28	51	54.9
PD-L1	11	50	22	5	57	8.8
PGP	3	63	4.8	1	79	1.3
PR	1	61	1.6	1	76	1.3
PTEN	68	71	95.8	79	86	91.9
RRM1	19	51	37.3	21	63	33.3
SPARCm	5	36	13.9	21	63	6.1
SPARCp	3	40	7.5	1	55	1.8
TLE3	21	61	34.4	25	76	32.9
TOP2A	37	62	59.7	47	78	60.3
**TOPO1**	45	71	63.4^*^	41	88	46.6
TS	38	70	54.3	49	84	58.3
**TUBB3**	61	67	91^*^	64	83	77.1

* indicates *P* value < 0.05;

^ indicates *P* value > 0.05 but < 0.10.

**Table 4:** Mutational analysis (sequencing): IDH wild type GBMs

	Patient age < 45			Patient age ≥ 70		
	Positive	Total tested	%	Positive	Total tested	%
ABL1	1	70	1.4	1	90	1.1
AKT1	0	72	0	1	95	1.1
ALK	0	72	0	0	95	0
Androgen Receptor	0	6	0	0	10	0
APC	3	72	4.2	7	95	7.4
ARAF	0	6	0	0	10	0
ATM	0	72	0	4	94	4.3
ATRX	2	5	40	1	9	11.1
BAP1	0	6	0	1	10	10
**BRAF**	9	72	12.5^*^	1	95	1.1
BRCA1	2	44	4.5	2	36	5.3
BRCA2	4	44	9.1	2	35	5.4
CDK4	0	6	0	0	10	0
CDKN2A	0	3	0	1	8	12.5
CHEK1	0	6	0	0	10	0
CHEK2	0	6	0	0	10	0
cKIT	5	72	6.9	2	95	2.1
cMET	1	72	1.4	0	95	0
CSF1R	2	72	2.8	0	95	0
CTNNB1	1	72	1.4	0	95	0
DDR2	0	6	0	0	10	0
EGFR	6	71	8.5	7	95	7.4
ERBB2	0	69	0	0	92	0
ERBB3	0	6	0	0	10	0
FGFR1	0	72	0	0	95	0
FGFR2	2	71	2.8	0	95	0
FGFR3	0	6	0	0	10	0
FLT3	0	71	0	1	95	1.1
GNA11	1	66	1.5	0	84	0
GNAQ	1	72	1.4	0	79	0
GNAS	0	62	0	0	95	0
HRAS	0	65	0	0	78	0
IDH1	0	72	0	0	95	0
IDH2	0	53	0	0	57	0
JAK2	0	72	0	1	95	1.1
KDR	0	72	0	1	95	1.1
KRAS	3	72	4.2	2	95	2.1
MEK1	0	6	0	0	10	0
MEK2	0	6	0	0	10	0
MLH1	2	71	2.8	0	95	0
MPL	0	71	0	0	92	0
NF1	4	6	66.7	2	10	20
NOTCH1	0	70	0	0	95	0
NRAS	0	72	0	1	95	1.1
NTRK1	0	6	0	0	10	0
**PDGFRA**	3	71	4.2^*^	0	94	0
PDGFRB	0	6	0	0	10	0
PIK3CA	6	72	8.3	6	95	6.3
PTCH1	0	6	0	1	10	10
PTEN	11	69	15.9	23	90	25.6
**PTPN11**	5	71	7^*^	1	95	1.1
RAF1	0	6	0	0	10	0
RET	0	66	0	2	88	2.3
ROS1	0	6	0	1	10	10
**SMARCA4**	3	6	50^*^	0	10	0
SMO	1	65	1.5	1	81	1.2
**TP53**	30	71	42.3^*^	23	94	24.5
VHL	0	65	0	0	88	0
WTI	0	6	0	1	10	10

* indicates *P* value < 0.05;

^ indicates *P* value > 0.05 but < 0.10.

To account for the possibly confounding issue of secondary versus primary GBM, we performed a sub-analysis to determine if there is an enrichment of secondary GBMs in our pediatric population. For this analysis we used EGFR expression and p53 mutation as surrogate markers for de novo and secondary GBM respectively. We did not find a significant association between EGFR expression and patient age in this sub-analysis. However, we did see significant enrichment of p53 mutations in younger patients, 57% [56/99] compared to 26% [25/97] in older patients (*P* < 0.0001). This analysis is present in Supplementary Table 1.

### MGMT promoter methylation incidence is higher in elderly patients

Pyro-sequencing was performed on 220 samples. MGMT promoter methylation was present in 41% (44/107) of younger patients and 49% (55/113) of older patients in histologically-defined GBM, suggesting a possible association of MGMT promoter methylation with an older cohort. When IDH wild-type GBM samples alone were analyzed, elderly patients showed significantly increased MGMT promoter methylation relative to younger patients (48% [43/89] versus 31% [21/6], respectively; *P* = 0.0334).

### Association between age and mutational frequency

**Figure 1:**
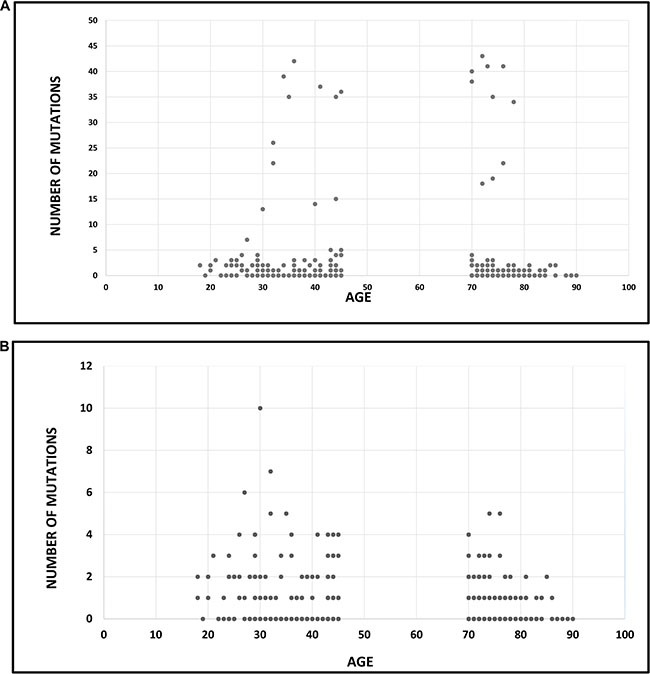
Association between age and mutational frequency in glioblastoma multiforme (**A**) Association between age and mutational load: analysis of 592 genes. The mean number of mutations was 3.44 (SD 9.4) for elderly patients and 3.49 (SD 8.165) for the younger patients (*P* = ns). (**B**) Association between age and mutational load: analysis of a panel of cancer-specific genes (*N* = 59). The mean number of mutations was 0.88 (SD 1.09) in elderly patients and 1.39 (SD 1.611) in younger patients (*P* = 0.004).

Evaluation of the entire database (592 genes) showed the mean number of mutations to be 3.44 (SD 9.4) for elderly patients and 3.49 (SD 8.165) for the younger patients, which was not found to be statistically significant by Cochran Armitage trend test (Figure 1A). Analysis of the panel of 59 confirmed cancer-related genes showed a significant difference in mutational frequency between young and elderly patients. Note that the selection of the 59 gene panel was based on research in literature, independent of the current study. The maximal number of mutations was 10, and were harbored by a young patient. The mean number of mutations in elderly patients was 0.88 (SD 1.09) compared with 1.39 (SD 1.611) in younger patients, and this was statistically significant by using Cochran Armitage trend test (*P* = 0.004) (Figure 1B).

## DISCUSSION

The standard of care for GBM is radiotherapy with concomitant and adjuvant temozolomide (TMZ). A notable proportion of GBM patients are resistant to current therapy, and durable control is generally elusive, leaving patients with limited therapeutic options. Additionally, the molecular heterogeneity of GBM poses a challenge in achieving therapeutic progress. Multiplatform tumor profiling can yield biomarker information that may prove clinically actionable and thereby potentially impacting clinical decision making. We performed a molecular analysis on a large cohort of GBM patients focusing on differential patterns of protein expression and genetic mutations seen in young and elderly patient cohorts. We found several molecular differences between age groups including enrichment of ALK, RRM1, and TUBB3 protein expression and *ATRX*, *BRAF*, *IDH1* and *TP53* mutations in the younger cohort. Our data suggest that the mutational landscape may actually be enriched in younger patients relative to older patients. As expected and consistent with the literature, *IDH1* and *ATRX* are more frequently mutated in younger patients, which confers a more favorable prognosis [17, 18]. There is currently active searches for candidate drugs via high-output screening of compounds and robust activity on developing agents targeting the IDH mutant [19, 20]. Our data suggests that screening for this target in a younger patient population may be more likely to yield candidates for trials that select based on IDH mutational status. Although molecular expression was different between young and elderly GBM patients, *BRAF* mutations overall were rare, constituting only 5.7% of patients. Nonetheless, younger patients are more likely to benefit from both *BRAF*- and *TP53-*targeted approaches [21, 22]. Interestingly, *PTEN* was the only gene to display increased mutation frequency in the elderly patient cohort. MGMT promoter methylation was also significantly higher in elderly patients (particularly *IDH* wild type) suggesting that the elderly may be more likely to benefit from a combination of TMZ and therapy targeting *PTEN* (i.e. PI3K/Akt/mTOR pathway). No differences in the immune checkpoint expression axis were found between young and elderly patients, and thus the benefit, or the lack thereof, is not predicted to be predicated on age.

The most frequently expressed protein in GBM was TUBB3, including in elderly patients. TUBB3 has been associated with multiple malignances and contributes to an aggressive phenotype, chemoresistance, and poor prognosis [23, 24]. GBMs are known to have alterations in the microtubule cytoskeleton and to exhibit cell motility and invasiveness. The expression of TUBB3 in GBM has been previously described, including an association with higher pathological grade [15, 25, 26]. However, an association of TUBB3 with younger age has not been previously reported and may be related to the role of TUBB3 in fetal central nervous system development [27]. Such robust expression in GBM makes TUBB3 a potential therapeutic target. Epothiolones have been shown to act on TUBB3-expressing glioma cells by inhibiting cell motility through cytoskeleton disruption, promoting tumor cell death by survivin down-regulation and by mediating therapeutic effects in preclinical models [28–30]. There have been several clinical trials dedicated to tubulin-targeted therapy for GBM, but results have been mixed and overall underwhelming [31–33]. The lack of therapeutic efficacy may be ascribed potentially to challenges with drug delivery as it remains unclear whether these agents have sufficient blood-brain barrier penetration. In addition, questions remain whether the target was successfully inhibited, or if the target was even present. Given the small numbers of subjects enrolled in these studies, if they contained an enrichment of elderly patients, the target may not have even been present.

RRM1, more frequently expressed in younger patients, is involved in DNA synthesis and repair [34, 35]. Low levels of RRM1 are associated with improved survival in patients with other types of malignancies treated with gemcitabine therapy [36, 37]. A recent phase I trial has been conducted to evaluate the tolerability of gemcitabine plus radiation therapy in patients with high-grade glioma [38]. Potentially, the responding patient population could be enriched by selecting for older patients or by screening for the lack of expression of RRM1.

Similar to TUBB3, ALK also has a role in embryonic central nervous system development [39] and is more frequently expressed in younger GBM patients. ALK is a receptor tyrosine kinase that binds to the growth factor pleiotrophin (PTN) [40] and has been previously shown to be expressed in GBM [15]. It has been implied that ALK contributes to gliomagenesis via multiple mechanisms including growth stimulation, mediation of anti-apoptotic pathways, self-renewal of glioma stem cells, and angiogenesis [40–43]. Furthermore, glioma patients with a higher expression of PTN or ALK have shorter survival times [39]. The ALK inhibitor Crizotinib has shown therapeutic effects in the GL261 model [43] and is currently in clinical trials for patients with lung cancer [44]. Our data suggest that a subset of young GBM patients may benefit from ALK-targeted therapy. Finally, our analysis indicates that a substantial proportion of GBM patients, regardless of age, may benefit from topoisomerase inhibitors (e.g., irinotecan, topotecan), which have been tested in GBM clinical trials in combination with other agents [45].

The current study employed a commercial database that is not clinically annotated regarding with reference to prior treatments therefore we are unable to account for the potentially confounding factor of treatment-induced mutational frequency [46]. It is likely that our cohort is enriched for posttreatment subjects since this is the usual scenario for comprehensive profiling when standard-of-care therapy has failed. In light of this, it should be noted that because as this is a commercial database, all findings may not be generalizable to the unselected GBM patient population. Furthermore, we are not able to correlate the molecular findings with clinical outcome. However as previously discussed, many of the differentially expressed alterations observed have previously been evaluated with regard to prognostic impact. Furthermore, because GBM samples were submitted at different stages of the disease-treatment process, we cannot relate molecular characteristics to tumor behavior. More importantly, we have very little data on the pattern of alterations present at GBM diagnosis, first recurrence, and subsequent recurrences. Because this is a clinical repository, such associations can't be determined. The genes included in the analysis were not randomly selected across the genome, which could also impact our conclusion that younger cases would have more mutations relative to a prior study [9] which has shown that mutation frequency increases with age, as has also been reported for other studies. Lastly, it is a fair consideration that the molecular differences between the young and elderly cohorts may reflect the difference between secondary and primary GBMs respectively. It is argued that due the molecular differences between them, they may represent completely different disease entities. We attempted to address issue this with a sub-analysis using surrogate makers of primary (EGFR expression) and secondary (p53 mutation) GBMs [47]. We found an increased frequency p53 mutations in our young patient population suggesting an enrichment of secondary GBMs. Despite these shortcomings, our study is strengthened by its size and the wide array of markers surveyed. In the case of GBM, most mutations are rare and will require large-scale comprehensive molecular profiling of a large number of patients in order to find enough patients with a specific actionable mutation(s) to determine efficacy of any targeted therapy. This study is the first to suggest that screening the tumors of younger patients for actionable targets may be more likely to reveal a biomarker result that is significant and influences clinical decision-making.

In summary, multiplatform tumor profiling has the potential to uncover biomarkers predictive of outcome and to potentially yield actionable clinical targets for therapy. By surveying this large cohort of GBM patients, we were able report significant molecular differences based on patient age. Interestingly, young patients showed a higher prevalence of key mutations and increased expression of two biomarkers, TUBB3 and ALK, which also have a known role in nervous system development. The enrichment of many GBM mutations in younger patients, with the sole exception of PTEN, offers many more therapeutic targets in contrast to the elderly population with GBM. Future studies will be focused on correlating large-scale molecular profiling data with radiographic tumor characteristics, tumor treatment response, and overall survival.

## MATERIALS AND METHODS

All samples were from patients with GBM (*N* = 375) and were submitted to Caris Life Science for multiplatform analysis between 2009 and 2016. Prior to analysis, the initial histological diagnosis was confirmed based on the World Health Organization (WHO) classification of 2007. Retrospective analysis was performed to identify biomarker characteristics of younger (< = 45 years) versus elderly (> = 70 years) cohorts of patients. Pediatric patients (< 18 years) were excluded. This study is exempt per policy 45 CFR 46.101 (b); the data analyzed is from an existing commercial repository and subject information is de-identified.

### Immunohistochemistry (IHC)

Analysis was performed on full formalin-fixed paraffin-embedded (FFPE) sections of GBM samples (*N* = 375) on glass slides. Slides were stained using automated staining techniques, per the manufacturer's instructions, and were optimized and validated per CLIA/CAO and ISO requirements. Inter-batch variability of the antibodies used was stringently monitored by a combination of western blotting against cell lysates and recombinant protein and liquid chromatography-tandem mass spectrometry (LC-MS/MS). Antibody batches that failed quality controls were eliminated. Tumor cells were scored for all proteins of interest with the exception of PD-1, which is scored on tumor infiltrating lymphocytes. Staining was scored for intensity (0 = no staining; 1+ = weak staining; 2+ = moderate staining; 3+ = strong staining) and staining percentage (0–100%). Results were categorized as positive or negative by defined thresholds specific to each marker based on published clinical literature that associates biomarker status with patient responses to therapeutic agents. A board-certified pathologist evaluated all IHC results independently.

### *In situ* hybridization

Detection of codeletion of chromosomes 1p and 19q 19q were assessed by fluorescence *in situ* hybridization (FISH) using the Abbott Molecular probes for 1p36/1q25 and 19p13/19q13. Codeletion was confirmed when ratios of 1p/1q signals and 19q/19p signals were both < 0.80. FISH or CISH was used to detect cMET gene amplification, and CISH was used to detect EGFR gene amplification.

### Fragment analysis (FA)

Fragment analysis (FA) was used to detect the EGFRvIII mutant. FA was performed on RNA extracted from FFPE samples. Two sets of flurophone-6-carboxy fluorescein (FAM)-linked primers were used in the PCR amplification of the wild-type and mutant EGFR alleles. PCR products were visualized using ABI-3500 × 1. Signal, generated from the wild-type alleles were used as an amplification control, and samples were considered positive if EGFRvIII was detected at a level 5x higher than the background signal.

### MGMT methylation testing

MGMT methylation testing was performed on DNA by pyro-sequencing-based analysis of 5CpG sites (CpGs 74–78). Samples with > 7% and < 9% methylation were considered equivocal and previously set cut points were used [48, 49].

### Mutational analysis

All patients’ samples did not undergo (next-generation sequencing) NGS, as data is collected from a commercial database comprising all GBM tumors that went through tumor profiling. At a given time, the ordering physician may have requested different tests for the patient, i.e., a full panel of tumor profiling versus a portion of a panel. Additionally, the cohort includes patients profiled before NGS was available. GBM samples were tested with NGS on genomic DNA isolated from FFPE tumor tissue. Among the 198 samples requested for analysis, 178 were sequenced using the Illumina MiSeq platform of which specific regions of 47 genes were amplified using the customized Illumina TruSeq Amplicon Cancer Hotspot panel. The remaining tumors (*N* = 20) were sequenced using the Illumina NextSeq platform on 592 genes. All variants were detected with > 99% confidence and with an analytical sensitivity of 5%.

A subset 59 genes (predetermined independently) was chosen for analysis because they are well-known cancer related genes, including known oncogenes, tumor suppressors, etc. This panel of 59 was interpreted by board-certified molecular geneticists and categorized as pathogenic, presumed pathogenic, variant of unknown significance, presumed benign, or benign, according to ACMG (American College of Medical Genetics and Genomics) standards, and results from both the Illumina MiSeq and the Illumina NextSeq platforms are included in the comparative analysis. Pathogenic, presumed pathogenic and variants of unknown significance were counted as mutations.

### Statistical analysis

Fisher's exact test was used to compare genetic and molecular mutation rates between two age groups (R v3.1.2). Cochran Armitage trend test [50] to examine whether the counts of mutations were different by age group. *P* values of <.05 were considered significant; a P of <.1 but >.05 was considered weakly significant. Multiple comparison adjustment was not conducted, owing to the exploratory nature of the study.

## SUPPLEMENTARY MATERIALS



## References

[1] Rudolph KL, Chang S, Lee HW, Blasco M, Gottlieb GJ, Greider C, DePinho RA (1999). Longevity, stress response, and cancer in aging telomerase-deficient mice. Cell.

[2] Stratton MR, Campbell PJ, Futreal PA (2009). The cancer genome. Nature.

[3] Tomasetti C, Vogelstein B (2015). Cancer etiology. Variation in cancer risk among tissues can be explained by the number of stem cell divisions. Science.

[4] Vogelstein B, Papadopoulos N, Velculescu VE, Zhou S, Diaz LA, Kinzler KW (2013). Cancer genome landscapes. Science.

[5] Snyder A, Makarov V, Merghoub T, Yuan J, Zaretsky JM, Desrichard A, Walsh LA, Postow MA, Wong P, Ho TS, Hollmann TJ, Bruggeman C, Kannan K (2014). Genetic basis for clinical response to CTLA-4 blockade in melanoma. N Engl J Med.

[6] Rizvi NA, Hellmann MD, Snyder A, Kvistborg P, Makarov V, Havel JJ, Lee W, Yuan J, Wong P, Ho TS, Miller ML, Rekhtman N, Moreira AL (2015). Cancer immunology. Mutational landscape determines sensitivity to PD-1 blockade in non-small cell lung cancer. Science.

[7] Curran WJ, Scott CB, Horton J, Nelson JS, Weinstein AS, Fischbach AJ, Chang CH, Rotman M, Asbell SO, Krisch RE, Nelson DF (1993). Recursive partitioning analysis of prognostic factors in three Radiation Therapy Oncology Group malignant glioma trials. J Natl Cancer Inst.

[8] Yabroff KR, Harlan L, Zeruto C, Abrams J, Mann B (2012). Patterns of care and survival for patients with glioblastoma multiforme diagnosed during 2006. Neuro Oncol.

[9] Kim H, Zheng S, Amini SS, Virk SM, Mikkelsen T, Brat DJ, Grimsby J, Sougnez C, Muller F, Hu J, Sloan AE, Cohen ML, Van Meir EG (2015). Whole-genome and multisector exome sequencing of primary and post-treatment glioblastoma reveals patterns of tumor evolution. Genome Res.

[10] Nobusawa S, Watanabe T, Kleihues P, Ohgaki H (2009). IDH1 mutations as molecular signature and predictive factor of secondary glioblastomas. Clin Cancer Res.

[11] Sanson M, Marie Y, Paris S, Idbaih A, Laffaire J, Ducray F, El Hallani S, Boisselier B, Mokhtari K, Hoang-Xuan K, Delattre JY (2009). Isocitrate dehydrogenase 1 codon 132 mutation is an important prognostic biomarker in gliomas. J Clin Oncol.

[12] Yan H, Parsons DW, Jin G, McLendon R, Rasheed BA, Yuan W, Kos I, Batinic-Haberle I, Jones S, Riggins GJ, Friedman H, Friedman A, Reardon D (2009). IDH1 and IDH2 mutations in gliomas. N Engl J Med.

[13] Hartmann C, Hentschel B, Wick W, Capper D, Felsberg J, Simon M, Westphal M, Schackert G, Meyermann R, Pietsch T, Reifenberger G, Weller M, Loeffler M (2010). Patients with IDH1 wild type anaplastic astrocytomas exhibit worse prognosis than IDH1-mutated glioblastomas, and IDH1 mutation status accounts for the unfavorable prognostic effect of higher age: implications for classification of gliomas. Acta Neuropathol.

[14] Ogura R, Tsukamoto Y, Natsumeda M, Isogawa M2, Aoki H2, Kobayashi T2, Yoshida S2, Okamoto K2, Takahashi H1, Fujii Y2, Kakita A1 (2015). Immunohistochemical profiles of IDH1, MGMT and P53: practical significance for prognostication of patients with diffuse gliomas. Neuropathology.

[15] Xiu J, Piccioni D, Juarez T, Pingle SC2, Hu J, Rudnick J, Fink K, Spetzler DB, Maney T, Ghazalpour A, Bender R, Gatalica Z, Reddy S (2016). Multi-platform molecular profiling of a large cohort of glioblastomas reveals potential therapeutic strategies. Oncotarget.

[16] Ha SY, Kang SY, Do IG, Suh YL (2013). Glioblastoma with oligodendroglial component represents a subgroup of glioblastoma with high prevalence of IDH1 mutation and association with younger age. J Neurooncol.

[17] Jiao Y, Killela PJ, Reitman ZJ, Rasheed AB, Heaphy CM, de Wilde RF, Rodriguez FJ, Rosemberg S, Oba-Shinjo SM, Nagahashi Marie SK, Bettegowda C, Agrawal N, Lipp E (2012). Frequent ATRX, CIC, FUBP1 and IDH1 mutations refine the classification of malignant gliomas. Oncotarget.

[18] Wiestler B, Capper D, Holland-Letz T, Korshunov A, von Deimling A, Pfister SM, Platten M, Weller M, Wick W (2013). ATRX loss refines the classification of anaplastic gliomas and identifies a subgroup of IDH mutant astrocytic tumors with better prognosis. Acta Neuropathol.

[19] Rohle D, Popovici-Muller J, Palaskas N, Turcan S, Grommes C, Campos C, Tsoi J, Clark O, Oldrini B, Komisopoulou E, Kunii K, Pedraza A, Schalm S (2013). An inhibitor of mutant IDH1 delays growth and promotes differentiation of glioma. Science.

[20] Brooks E, Wu X, Hanel A, Nguyen S, Wang J, Zhang JH, Harrison A, Zhang W (2014). Identification and characterization of small-molecule inhibitors of the R132H/R132H mutant isocitrate dehydrogenase 1 homodimer and R132H/wild-type heterodimer. J Biomol Screen.

[21] Nicolaides TP, Li H, Solomon DA, Hariono S, Hashizume R, Barkovich K, Baker SJ, Paugh BS, Jones C, Forshew T, Hindley GF, Hodgson JG, Kim JS (2011). Targeted therapy for BRAFV600E malignant astrocytoma. Clin Cancer Res.

[22] Dasgupta T, Olow AK, Yang X, Hashizume R, Nicolaides TP, Tom M, Aoki Y, Berger MS, Weiss WA, Stalpers LJ, Prados M, James CD, Mueller S (2016). Survival advantage combining a BRAF inhibitor and radiation in BRAF V600E-mutant glioma. J Neurooncol.

[23] Koh Y, Jang B, Han SW, Kim TM, Oh DY, Lee SH, Kang CH, Kim DW, Im SA, Chung DH, Kim YT, Kim TY, Kim YW (2010). Expression of class III beta-tubulin correlates with unfavorable survival outcome in patients with resected non-small cell lung cancer. J Thorac Oncol.

[24] McCarroll JA, Sharbeen G, Liu J, Youkhana J, Goldstein D, McCarthy N, Limbri LF, Dischl D, Ceyhan GO, Erkan M, Johns AL, Biankin AV, Kavallaris M (2015). βIII-tubulin: a novel mediator of chemoresistance and metastases in pancreatic cancer. Oncotarget.

[25] Katsetos CD, Dráberová E, Smejkalová B, Reddy G, Bertrand L, de Chadarévian JP, Legido A, Nissanov J, Baas PW, Dráber P (2007). Class III beta-tubulin and gamma-tubulin are co-expressed and form complexes in human glioblastoma cells. Neurochem Res.

[26] Katsetos CD, Del Valle L, Geddes JF, Assimakopoulou M, Legido A, Boyd JC, Balin B, Parikh NA, Maraziotis T, de Chadarevian JP, Varakis JN, Matsas R, Spano A (2001). Aberrant localization of the neuronal class III-tubulin in astrocytomas: a marker for anaplastic potential. Arch Pathol Lab Med.

[27] Katsetos CD, Legido A, Perentes E, Mörk SJ (2003). Class III beta-tubulin isotype: a key cytoskeletal protein at the crossroads of developmental neurobiology and tumor neuropathology. J Child Neurol.

[28] Dietzmann A, Kanakis D, Kirches E, Kropf S, Mawrin C, Dietzmann K (2003). Nanomolar concentrations of epothilone D inhibit the proliferation of glioma cells and severely affect their tubulin cytoskeleton. J Neurooncol.

[29] Quick QA (2008). Epothilone B induces glioblastoma cell death via survivin down-regulation. Exp Oncol.

[30] O'Reilly T, Wartmann M, Maira SM, Hattenberger M, Vaxelaire J, Muller M, Ferretti S, Buchdunger E, Altmann KH, McSheehy PM (2005). Patupilone (epothilone B, EPO906) and imatinib (STI571, Glivec) in combination display enhanced antitumour activity *in vivo* against experimental rat C6 glioma. Cancer Chemother Pharmacol.

[31] Peereboom DM, Supko JG, Carson KA, Batchelor T, Phuphanich S, Lesser G, Mikkelsen T, Fisher J, Desideri S, He X, Grossman SA (2010). New Approaches to Brain Tumor Therapy (NABTT) Consortium. A phase I/II trial and pharmacokinetic study of ixabepilone in adult patients with recurrent high-grade gliomas. J Neurooncol.

[32] Stupp R, Tosoni A, Bromberg JE, Hau P, Campone M, Gijtenbeek J, Frenay M, Breimer L, Wiesinger H, Allgeier A, van den Bent MJ, Bogdahn U, van der Graaf W (2011). Sagopilone (ZK-EPO, ZK 219477) for recurrent glioblastoma. A phase II multicenter trial by the European Organisation for Research and Treatment of Cancer (EORTC) Brain Tumor Group. Ann Oncol.

[33] Oehler C, Frei K, Rushing EJ, McSheehy PM, Weber D, Allegrini PR, Weniger D, Lütolf UM, Knuth A, Yonekawa Y, Barath K, Broggini-Tenzer A, Pruschy M (2012). Patupilone (epothilone B) for recurrent glioblastoma: clinical outcome and translational analysis of a single-institution phase I/II trial. Oncology.

[34] lledge SJ, Zhou Z, Allen JB (1992). Ribonucleotide reductase: Regulation, regulation, regulation. Trends Biochem Sci.

[35] Reichard P (1993). From RNA to DNA, why so many ribonucleotide reductases?. Science.

[36] Reynolds C, Obasaju C, Schell MJ, Li X, Zheng Z, Boulware D, Caton JR, Demarco LC, O'Rourke MA, Shaw Wright G, Boehm KA, Asmar L, Bromund J (2009). Randomized phase III trial of gemcitabine-based chemotherapy with *in situ* RRM1 and ERCC1 protein levels for response prediction in non-small-cell lung cancer. J Clin Oncol.

[37] Simon GR, Schell MJ, Begum M, Kim J, Chiappori A, Haura E, Antonia S, Bepler G (2012). Preliminary indication of survival benefit from ERCC1 and RRM1-tailored chemotherapy in patients with advanced nonsmall cell lung cancer: evidence from an individual patient analysis. Cancer.

[38] Kim MM, Camelo-Piragua S, Schipper M, Tao Y, Normolle D, Junck L, Mammoser A, Betz BL, Cao Y, Kim CJ, Heth J, Sagher O, Lawrence TS (2016). Gemcitabine plus radiation therapy for high-grade glioma: long-term results of a phase 1 dose-escalation study. Int J Radiat Oncol Biol Phys.

[39] Wellstein A (2012). ALK receptor activation, ligands and therapeutic targeting in glioblastoma and in other cancers. Front Oncol.

[40] Stylianou DC, Auf der Maur A, Kodack DP, Henke RT, Hohn S, Toretsky JA, Riegel AT, Wellstein A (2009). Effect of single-chain antibody targeting of the ligand-binding domain in the anaplastic lymphoma kinase receptor. Oncogene.

[41] Bowden ET, Stoica GE, Wellstein A (2002). Anti-apoptotic signaling of pleiotrophin through its receptor, anaplastic lymphoma kinase. J Biol Chem.

[42] Koyama-Nasu R, Haruta R, Nasu-Nishimura Y, Taniue K, Katou Y, Shirahige K, Todo T, Ino Y, Mukasa A, Saito N, Matsui M, Takahashi R, Hoshino-Okubo A (2014). The pleiotrophin-ALK axis is required for tumorigenicity of glioblastoma stem cells. Oncogene.

[43] Zhang L, Kundu S, Feenstra T, Li X, Jin C, Laaniste L, El Hassan TE, Ohlin KE, Yu D, Olofsson T, Olsson AK, Pontén F, Magnusson PU (2015). Pleiotrophin promotes vascular abnormalization in gliomas and correlates with poor survival in patients with astrocytomas. Sci Signal.

[44] Solomon BJ, Mok T, Kim DW, Wu YL, Nakagawa K, Mekhail T, Felip E, Cappuzzo F, Paolini J, Usari T, Iyer S, Reisman A, Wilner KD (2014). First-line crizotinib versus chemotherapy in ALK-positive lung cancer. N Engl J Med.

[45] Reardon DA, Quinn JA, Rich JN, Desjardins A, Vredenburgh J, Gururangan S, Sathornsumetee S, Badruddoja M, McLendon R, Provenzale J, Herndon JE, Dowell JM, Burkart JL (2005). Phase I trial of irinotecan plus temozolomide in adults with recurrent malignant glioma. Cancer.

[46] Johnson BE, Mazor T, Hong C, Barnes M, Aihara K, McLean CY, Fouse SD, Yamamoto S, Ueda H, Tatsuno K, Asthana S, Jalbert LE, Nelson SJ (2014). Mutational analysis reveals the origin and therapy-driven evolution of recurrent glioma. Science.

[47] Crespo I, Vital AL, Gonzalez-Tablas M, Patino Mdel C, Otero A, Lopes MC, de Oliveira C, Domingues P, Orfao A, Tabernero MD (2015). Molecular and Genomic Alterations in Glioblastoma Multiforme. Am J Pathol.

[48] Hegi ME, Diserens AC, Gorlia T, Hamou MF, de Tribolet N, Weller M, Kros JM, Hainfellner JA, Mason W, Mariani L, Bromberg JE, Hau P, Mirimanoff RO (2005). MGMT gene silencing and benefit from temozolomide in glioblastoma. N Engl J Med.

[49] Quillien V, Lavenu A, Karayan-Tapon L, Carpentier C, Labussière M, Lesimple T, Chinot O, Wager M, Honnorat J, Saikali S, Fina F, Sanson M, Figarella-Branger D (2012). Comparative assessment of 5 methods (methylation-specific polymerase chain reaction, MethyLight, pyrosequencing, methylation-sensitive high-resolution melting, and immunohistochemistry) to analyze O6-methylguanine-DNA-methyltranferase in a series of 100 glioblastoma patients. Cancer.

[50] Agresti A (2012). Categorical Data Analysis.

