# Supplementary material for: GBM-associated mutations and altered protein expression are more common in young patients
Source: Oncotarget. 2016 Aug 25;7(43):69466–78. doi: 10.18632/oncotarget.11617 (PMC5342491; doi:10.18632/oncotarget.11617)
Supplement: Supplementary file 1 [file oncotarget-07-69466-s001.pdf]

GBM-associated mutations and altered protein expression are more common in young patients

Supplementary Materials

Supplementary Table S1: EGFR expression and p53 mutation frequency in young and elderly patients

Age Group	EGFR expression	p53 mutation
Old	45% ($n = 42$)	26% ($n = 25$)
Young	55% ($n = 51$)	57% ($n = 56$)
	p = NS	$p < 0.0001$
